# Ventriculoperitoneal Shunt Tap Task Trainer: A Technical Report

**DOI:** 10.7759/cureus.41307

**Published:** 2023-07-03

**Authors:** Jillian Connors, Andrew Kobets, Orna Rosen

**Affiliations:** 1 Division of Neonatology, The Children's Hospital at Montefiore, Bronx, USA; 2 Division of Neurosurgery, The Children's Hospital at Montefiore, Bronx, USA

**Keywords:** constructing low-cost relatively high-fidelity task trainers, cerebrospinal fluid (csf), ventriculoperitoneal shunt complications/malfunction, ventriculoperitoneal shunts, pediatric hydrocephalus

## Abstract

This technical report describes the creation of a model of an infant with a ventriculoperitoneal shunt (VPS). This model is authentic, assembled easily, and reusable which allows for pediatric and neurosurgical practitioners to gain experience in performing VPS taps. Learning objectives have been provided to guide task training.

## Introduction

Hydrocephalus is caused by an imbalance in cerebrospinal fluid (CSF) production, flow, and reabsorption and results in ventriculomegaly, macrocephaly, and/or increased intracranial pressure (ICP) [1]. It is categorized as either congenital or acquired. Causes of congenital hydrocephalus include aqueductal stenosis, Chiari Type II malformation associated with myelomeningocele, and a variety of genetic syndromes. Acquired hydrocephalus may result from intrauterine viral infections, intraventricular hemorrhages, tumors, or trauma. Increased ICP and progressive ventriculomegaly may worsen in congenital and acquired hydrocephalus, result in damage to neural tissues, and are generally treated with CSF diversion [2,3]. While many patients will undergo definitive treatment with ventriculoperitoneal shunt (VPS) placement, some patients, such as small or critically ill infants, will require a temporizing ventricular access device such as an Ommaya reservoir.

VPS and ventricular access devices carry risks of malfunction and infection. When these complications occur, VPS tapping is considered to evaluate shunt function and perform CSF analysis. However, performing a VPS tap itself can introduce infection into the access port or air into the system resulting in obstruction, particularly if performed by an inexperienced practitioner [4]. Because VPS tapping may be an uncommon or infrequent procedure in some neonatal and pediatric intensive care units, developing a task trainer and utilizing simulation allow pediatric and neurosurgical practitioners to achieve procedural competency. Our group has published previously on the development of authentic, inexpensive, and easily modifiable task trainers that may be used to increase procedural competency and confidence in the NICU [5-9].

## Technical report

**Figure 1:**
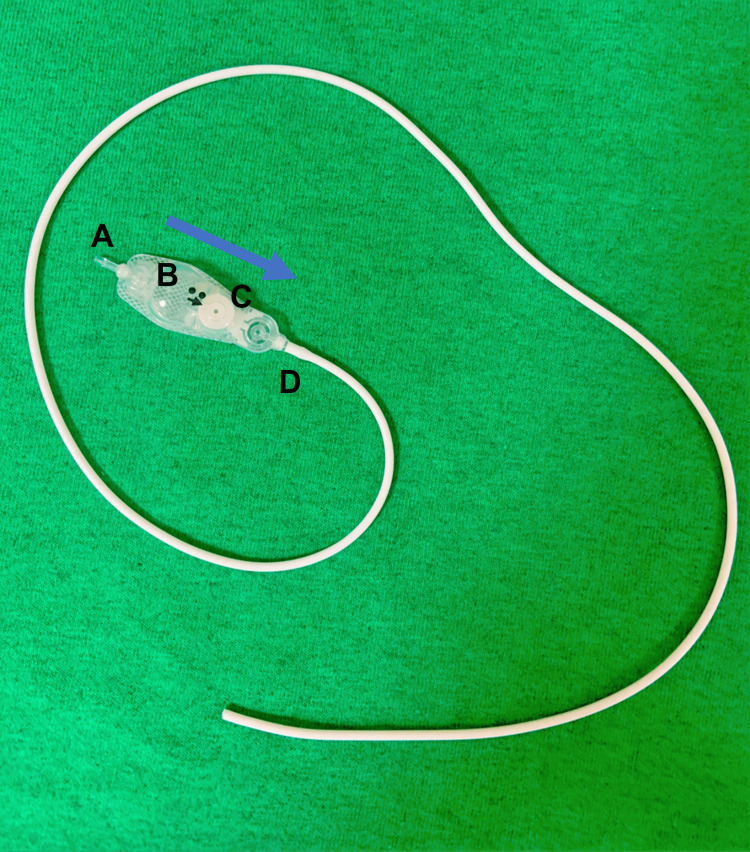
Medtronic Strata II programable shunt valve. Cerebrospinal fluid (arrow) flows into the shunt via the ventricular port (A), through the reservoir (B), the valve mechanism (C), and the peritoneal port (D). The peritoneal catheter is attached to the peritoneal port (D).

To create this VPS task trainer, we modified a low-fidelity Simulaids neonatal resuscitation manikin (Simulaids Ltd, United Kingdom). For manikin modification, we used a Medtronic Strata II programmable shunt valve and peritoneal catheter (Medtronic PS Medical, Inc, Minneapolis, MN), a bulb syringe, and water to simulate CSF (Figure 1).

**Figure 2:**
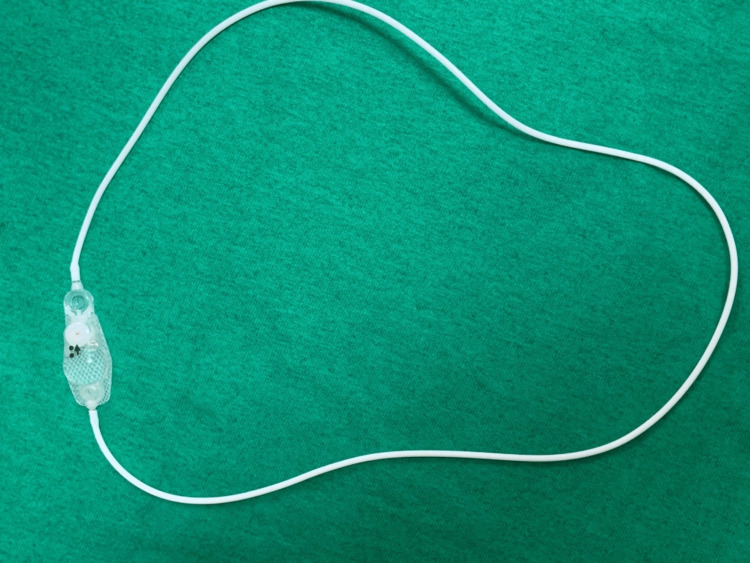
The peritoneal catheter is attached at both the ventricular and peritoneal ports creating a closed loop.

The peritoneal catheter was attached to both the ventricular port and the peritoneal port, creating a closed circuit (Figure 2).

**Figure 3:**
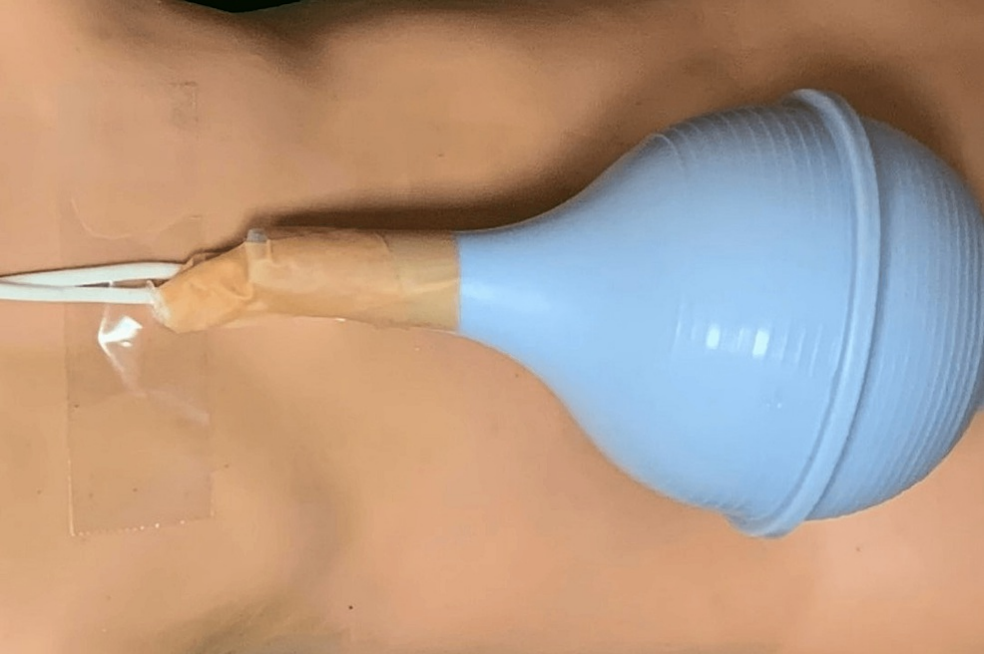
The proximal and distal aspects of the peritoneal catheter are secured into the bulb syringe using zinc oxide-based waterproof tape.

The peritoneal catheter was then cut equidistant from the ventricular and peritoneal ports. The bulb syringe was prepared by cutting approximately 2cm from the tip and was filled with 60ml of water. If desired, 1-2ml of povidone-iodine may be added to give the water a yellow color. The distal piece of the peritoneal catheter was placed into the bulb syringe. With a water-filled 5ml syringe attached to a 24-gauge needle, additional water was instilled into the proximal (ventricular) part of the peritoneal catheter and shunt reservoir. The proximal part of the ventricular catheter was then placed into the bulb syringe. Both catheters were secured to the bulb syringe with zinc oxide-based waterproof tape, although any adhesive tape may be used (Figure 3).

**Figure 4:**
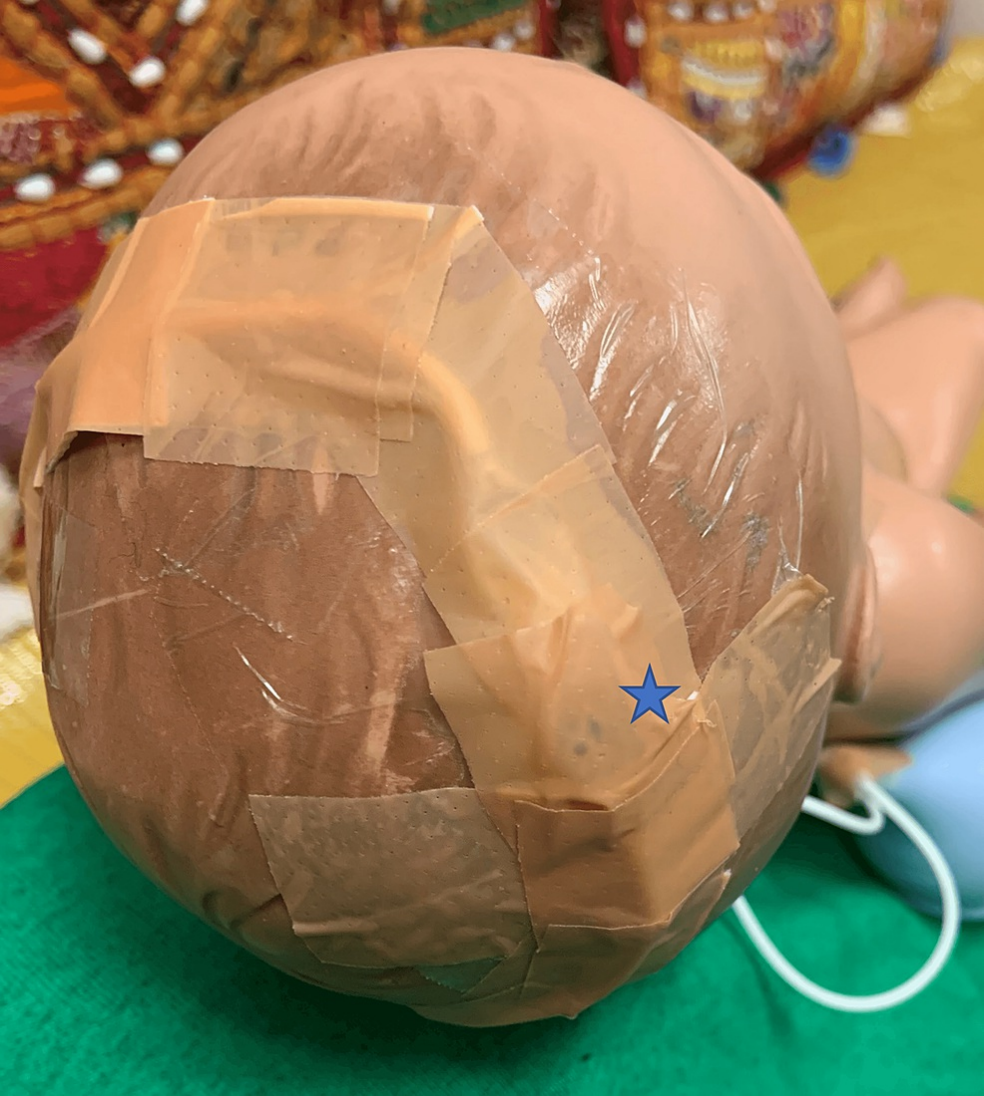
Ventriculoperitoneal shunt reservoir secured to the manikin’s right parietal scalp (star), and both parts of the peritoneal catheter are secured around the crown of the manikin’s scalp with zinc oxide-based waterproof tape.

The VPS reservoir was then secured to the manikin’s right parietal scalp using zinc oxide-based tape. Both parts of the peritoneal catheter were secured around the crown of the manikin’s head with tape (any adhesive tape may be used) (Figure 4).

**Figure 5:**
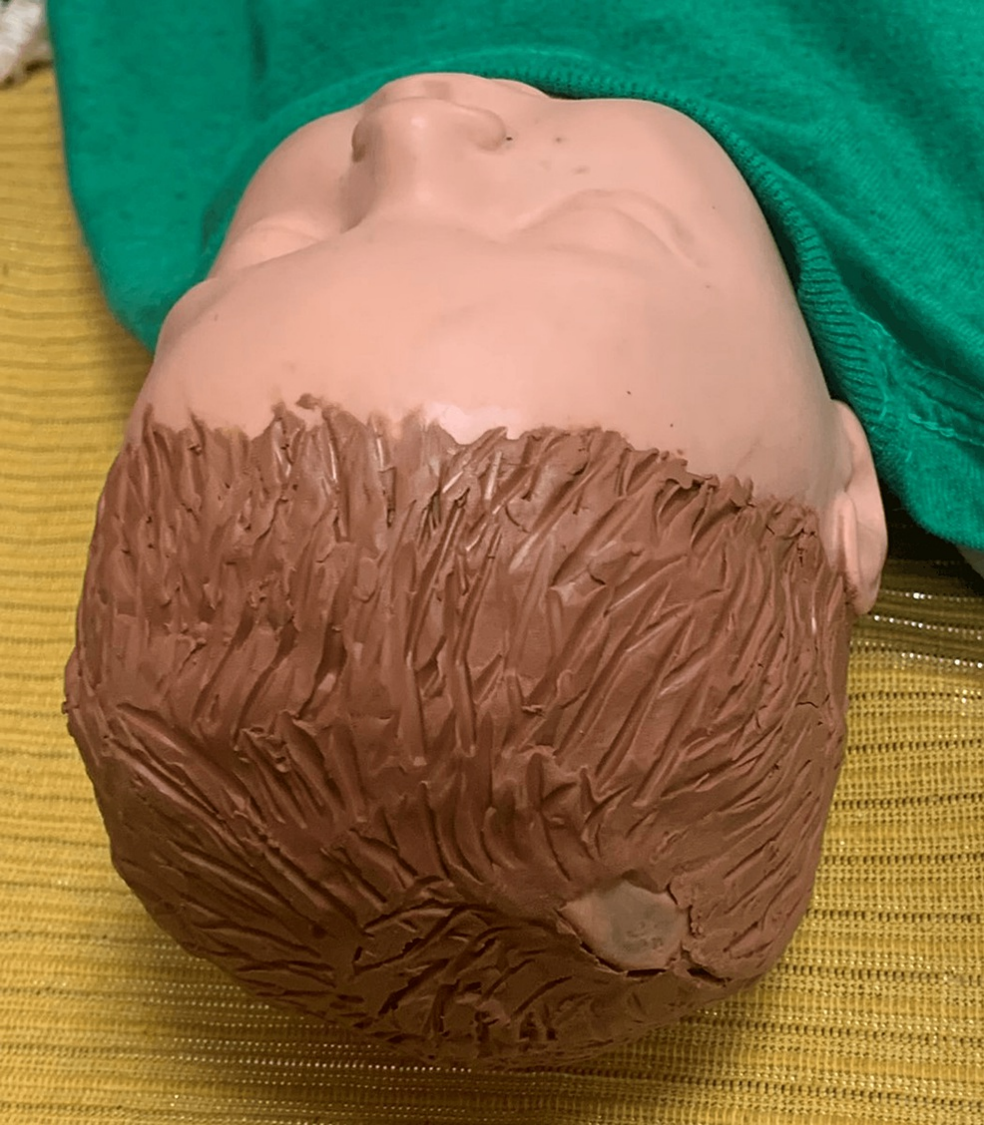
Modeling clay placed over the scalp except the area overlying the ventriculoperitoneal shunt reservoir.

If desired, modeling clay can be used to cover all areas of the scalp except the area overlying the VPS reservoir giving the appearance of hair for additional authenticity (Figure 5).

Technique

The VPS tapping procedure was adapted from MacDonald’s Atlas of Procedures in Neonatology [10]. Equipment needed for VPS tap is provided (Table 1).

**Table 1:** Additional equipment needed for ventriculoperitoneal shunt tap.

Additional Equipment	
Sterile gloves, gowns, and drapes	25-gauge butterfly needle
Masks and surgical/bouffant caps	5 milliliter syringes
Povidone-iodine or chlorohexidine	Specimen containers for cerebrospinal fluid analysis
Sterile gauze	Adhesive bandage
Optional	
Manometer	Topical lidocaine cream

**Figure 6:**
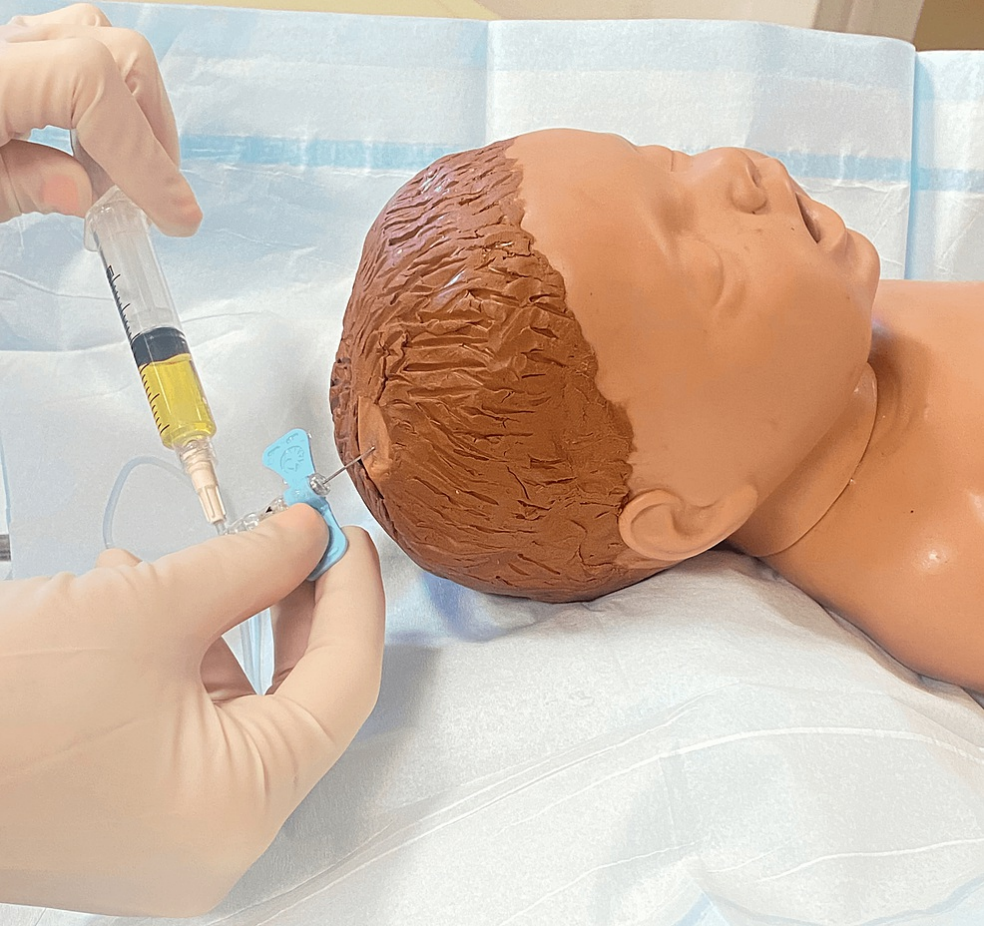
Aspiration of cerebrospinal fluid from the shunt reservoir.

As needed, sedation or topical anesthetic is provided to the patient. The patient is then placed in the supine position and is gently restrained. Infants, for example, may be swaddled in a developmentally appropriate manner. The patient’s head is turned to demonstrate the shunt valve. The skin overlying the shunt reservoir is prepped with povidone-iodine or chlorohexidine. A 25-gauge butterfly needle is then inserted at a 45-degree angle into the reservoir. Spontaneous CSF egress should be noted (Figure 6).

If opening pressure measurement is indicated, a manometer can be attached. CSF is then sampled no faster than 1ml per minute. The total CSF volume removed should not exceed 10ml per kg. If CSF analysis is needed, a specimen may be sent for cell count, glucose, protein, and bacterial culture. Next, distal flow through the VPS valve and distal tubing may be assessed by depressing the proximal catheter with a sterile finger to prevent CSF flow into the ventricular system, and gently injecting 3ml of aspirated CSF. If there is distal obstruction, CSF will not be able to be injected into the system and resistance will be met. In cases of partial or unstable distal obstruction, there may be initial resistance that is relieved when the obstruction is dislodged followed by free flow of CSF. If there is concern or radiographic evidence of distal catheter kinking or disconnection, assessment of distal flow should not be performed as it may deposit CSF in the subcutaneous tissue or give a false sense of appropriate distal flow. At completion of the procedure, the needle is removed, and pressure is held over the site for approximately two minutes. An adhesive bandage is then applied to the procedural site. The patient should be monitored post-procedure for any changes in neurologic exam.

## Discussion

The model we have created has several benefits. The modifications are simple to create and assemble. The task trainer is also inexpensive. We utilized an older, low-fidelity manikin that had been replaced by newer models in our simulation program. In addition, we used a VPS device with a lapsed expiration date that would not be able to be used in patient care but that replicates what is encountered in clinical practice. The model is lightweight and portable, allowing for simulation to be done in situ. Last, the task trainer is reusable. This allows for many learners, including residents, fellows, advanced practice providers (nurse practitioners and physician assistants), and even attending physicians, to practice and gain procedural competency. Learning objectives for task training are provided in the appendix. Based on our review of the literature, while simulation is frequently used for complex neurosurgical procedures, an authentic, easily assembled clinical VPS task trainer has not been described [11,12].

The use of this task trainer has been incorporated into the neonatal-perinatal fellow simulation curriculum and NICU nurse practitioner and physician assistant continuing education at our institution. At the completion of the simulation sessions, neonatal fellows and advanced practice providers are directed to debrief the medical and technical aspects of the simulation. Neonatal-perinatal medicine and neurosurgical faculty facilitate the debrief, reinforcing the indications for and technique needed for performing VP shunt taps. Feedback from NICU fellows and neonatal advanced practice providers elicited through facilitated debriefing has been positive; they have been successful in achieving the learning objectives outlined in the appendix and expressed feeling more confident in performing the procedure. In addition, during durability testing, the task trainer continued to function reliably after more than 10 consecutive tapping procedures.

Incorporating this task trainer into an existing simulation curriculum will allow not only for assessment and attainment of procedural competency, but also provides a unique tool that can be utilized when reviewing hydrocephalus pathophysiology, highlighting the indications for, benefits, risks, and alternatives of the VPS tapping procedure and post-procedure complications, such as hemorrhage, hemodynamic instability, and electrolyte abnormalities. In addition to VPS simulation, this model and the technique described above may also be used for cases of ventricular access device tapping. A scenario of a preterm infant with posthemorrhagic hydrocephalus who requires an Ommaya reservoir tap is one example.

While there is concern that performing VPS taps may introduce infection into the system, the literature suggests that this risk is actually very low when performed either by neurosurgical or NICU practitioners [13,14]. This highlights the importance of using maximally aseptic precautions and is why we perform the task training procedure using a sterile technique.

This task trainer may be modified to utilize other shunt systems that are commonly used at other institutions. It can also be modified using smaller, more complex valve systems. This will add an additional degree of difficulty for learners that mirrors what is seen in clinical practice. At our institution, this task trainer will be incorporated into the neurosurgical resident curriculum. In addition, future work will utilize this task trainer to better quantify shunt tapping procedural performance and competency.

## Conclusions

The modification of a low-fidelity neonatal resuscitation manikin described in this technical report is simple, inexpensive, and reusable allowing many pediatric and neurosurgical trainees to gain procedural competency and increase their comfort in performing VPS taps. It is also authentic, and skills obtained using this task trainer directly translate to the bedside. VPS tapping may only occur rarely in some neonatal and pediatric intensive care units, so having a task trainer available will permit providers to gain valuable experience, confidence, and comfort in performing this procedure in a low-risk environment. Additionally, this task trainer can be utilized in existing neonatal, pediatric, and neurosurgical simulation curricula to add complexity to existing scenarios and broaden the education of learners at many different levels of training and experience.

## Appendices

Learning Objectives

1. Describe the indications for ventriculoperitoneal shunt tapping.

2. Gather supplies needed for ventriculoperitoneal shunt tapping.

3. Correctly perform ventriculoperitoneal shunt tap.
